# Decoding the impact of lipid saturation on ER signaling networks, ERSU, UPR, and ERAD

**DOI:** 10.1371/journal.pone.0345910

**Published:** 2026-08-14

**Authors:** Xia Li, Maho Niwa

**Affiliations:** Department of Molecular Biology, School of Biological Sciences, University of California, San Diego, California, United States of America; University of Pittsburgh, UNITED STATES OF AMERICA

## Abstract

The faithful inheritance of a functional endoplasmic reticulum (ER) in *Saccharomyces cerevisiae* is safeguarded by the ER Stress Surveillance (ERSU) checkpoint, which delays cytokinesis when ER homeostasis is perturbed. Under stress, ER transmission to the daughter cell is halted, while in parallel—but through independent pathways—the Unfolded Protein Response (UPR) restores ER function and ER-associated degradation (ERAD) eliminates misfolded proteins, ultimately allowing cell cycle re-entry. ER stress also transiently stimulates sphingolipid biosynthesis, with the intermediate phytosphingosine (PHS) acting as a key activator of ERSU. Yet how broader lipid parameters—such as membrane composition and saturation—reshape ER quality control and, in particular, govern ER inheritance during division remains poorly understood. To begin addressing this question, a tightly controlled experimental system was employed to selectively alter lipid saturation while monitoring ER inheritance within the context of ER homeostasis maintained by the UPR and ERAD. This analysis revealed that perturbations in lipid saturation exert specific effects on ER inheritance that are distinct from their impact on UPR activation and ERAD efficiency. These findings support a central role for lipid homeostasis in ER functional regulation and suggest that membrane lipid composition contributes to the coordination of ERSU, UPR, and ERAD during ER inheritance under stress.

## Introduction

In eukaryotic cells, essential biochemical reactions are compartmentalized within membrane-bound organelles, which provides spatial and regulatory control over diverse cellular processes. Among these organelles, the endoplasmic reticulum (ER) stands out as one of the largest and most functionally diverse. It acts as a central hub for the synthesis, folding, and maturation of proteins destined for secretion or membrane insertion—collectively classified as secretory pathway proteins [1–7]. These proteins are assisted by molecular chaperones to ensure correct folding and undergo tightly regulated post-translational modifications, including glycosylation, disulfide bond formation, and oligomerization, before being exported to the Golgi apparatus and beyond.

The ER is also the principal site of lipid biosynthesis, generating a broad spectrum of phospholipids, sterols, and lipid-derived molecules that are essential for the structural integrity and functional capacity of intracellular membranes. Beyond serving as the structural matrix of cellular compartments, lipids tune membrane properties such as curvature, fluidity, and thickness—parameters that in turn influence membrane protein folding, insertion, and trafficking. Studies in yeast and mammalian systems has underscored the intimate link between ER lipid composition and ER function: imbalances in lipid saturation or phospholipid availability can impair protein folding, alter membrane protein dynamics, and disrupt ER morphology [8–12]. Elevated levels of saturated fatty acids (SFA), particularly in mammalian cells, decrease membrane flexibility, perturb lipid–protein interactions, and induce defects in vesicle trafficking, calcium signaling, and protein quality control [11–14]. Conversely, perturbations in specific lipid species—such as phosphatidylinositol depletion in yeast—compromise ER function by disturbing lipid-dependent signaling pathways, membrane properties, and organelle communication [12,13]. The ER also serves as a major calcium storage organelle, and its ability to coordinate calcium homeostasis is tightly coupled to lipid composition, further shaping cell signaling and metabolic outputs [15,16].

Given its diverse physiological roles, the ER must continually adapt to environmental and metabolic changes while preserving homeostasis. Perturbations such as protein misfolding or lipid imbalance induce ER stress and activate a suite of adaptive quality control pathways that act cooperatively to restore ER function and limit cellular damage [11,12]. These events are further coordinated with the cell cycle to ensure accurate ER inheritance. In the budding yeast *Saccharomyces cerevisiae*, the ER is partitioned into two major domains: the perinuclear ER (pnER), which envelops the nucleus, and the cortical ER (cER), which forms a dynamic network beneath the plasma membrane. Because the ER cannot be synthesized *de novo* during cell division, it must be faithfully inherited from the mother cell. Under the normal growth, ER inheritance begins with the formation of an initial ER tubule (IET) that emerges from the pnER and extends toward the bud in early stages of the cell cycle, later expanding into a functional cER network in the daughter cell. Accurate inheritance is critical, as failures in this process compromise ER function in daughter cells and can lead to loss of viability [17,18].

To safeguard ER integrity and function during stress, eukaryotic cells rely on three major partially overlapping pathways: the ER Stress Surveillance (ERSU) pathway, ER-associated degradation (ERAD), and the unfolded protein response (UPR). Together, these pathways sense and respond to distinct but interconnected forms of ER dysfunction, forming a coordinated and robust quality control network [6,7,19–28]. In yeast, ERSU functions as a stress-activated cell cycle checkpoint that halts ER inheritance during mitosis; when ER stress is detected, ERSU blocks cER inheritance and cytokinesis, thereby preventing the transmission of damaged ER to the daughter cell and preserving daughter cell viability [18,29]. ERAD maintains proteostasis by recognizing misfolded proteins within the ER, retro-translocating them to the cytosol, and targeting them for destruction by the ubiquitin–proteasome system [21,28]. Misfolded substrates are classified according to the location of the defective domain—luminal (ERAD-L), membrane (ERAD-M), or cytosolic (ERAD-C)—and are processed by distinct ubiquitin ligase complexes, such as Hrd1 and Doa10, in conjunction with the Cdc48 ATPase [30,31]. Multiple studies have shown that lipid perturbations—including increased lipid saturation or depletion of key phospholipids—can modulate ERAD efficiency and substrate selectivity, linking membrane status to protein quality control [8,11,12,32,33]. The third major pathway, the UPR, responds to ER stress by inducing broad transcriptional and translational reprogramming that boosts ER folding capacity and expands the ER network. In yeast, UPR activation is initiated by the ER-resident sensor Ire1, which catalyzes unconventional splicing of HAC1 mRNA, producing the Hac1 transcription factor that upregulates genes involved in protein folding, ERAD, and lipid biosynthesis [34]. Moreover, emerging evidence indicates that lipid abnormalities—such as increased membrane saturation or impaired phospholipid synthesis—can activate the UPR independently of overt protein misfolding, enabling Ire1 to integrate proteotoxic and lipotoxic signals to gauge ER homeostasis [35].

Despite these advances, it remains unclear how different forms of lipid imbalance shape the magnitude, duration, and coordination of ER stress response pathways. Extensive work has defined the individual roles and mechanisms of ERSU, ERAD, and UPR in responding to classical protein misfolding stresses, yet the integrated behavior of these pathways under conditions of membrane lipid imbalance is still poorly understood. In particular, how perturbations in membrane composition—such as altered lipid saturation or changes in phospholipid flux—impact ER inheritance, protein degradation, and stress signaling thresholds has not been systematically explored.

To address these gaps, a multifaceted strategy was used to manipulate ER lipid composition and assess the consequences for ER quality control. First, a previously established experimental system was employed [36] to gradually increase ER membrane lipid saturation, enabling analysis of how progressive changes in membrane properties affect ER function. In parallel, ER homeostasis was examined under inositol depletion, a condition that disrupts phosphatidylinositol synthesis and remodels the cellular phospholipid pool, thereby imposing a distinct form of lipid stress without necessarily increasing saturation.

Using these complementary perturbations, the effects of altered lipid homeostasis on each of the major ER stress response pathways—ERSU, ERAD, and UPR—were systematically evaluated. The findings reveal that increased membrane saturation and phospholipid imbalance impair cER inheritance, lower the threshold for UPR activation, and selectively disrupt ERAD processing of membrane-associated substrates. These results support a central role for ER membrane composition in regulating ER quality control pathways and, more broadly, highlight how lipid–protein interactions shape cellular responses to stress, linking membrane biology to proteostasis and cell cycle progression.

## Materials and methods

### Yeast and plasmids methods

Yeast cultures were grown at 30 °C in standard growth medium as previously described [37]. Plasmids were introduced into yeast using the lithium acetate transformation method [38]. A complete list of yeast strains and plasmids used in this study is provided in S1 and S2 Tables, respectively. Gene deletions and epitope tagging were performed using PCR-based homologous recombination as described [39]. Plasmids pRH469, pRH1377, pRH2058, and pRH2997 were generously provided by the Randy Hampton lab. Plasmids pRH1377, pRH2058, or pRH2997 were directly transformed into the indicated yeast strains. Plasmid pRH469 was linearized with StuI and integrated into the URA3 locus of the specified strains. ER localization was monitored using Pho88-GFP, a GFP-tagged ER membrane protein [40].

For inositol depletion experiments, yeast strains were initially grown on inositol-containing plates. Cells were then cultured overnight in inositol-free medium (prepared using yeast nitrogen base without amino acids and inositol) to reach mid-exponential phase. Cultures were freshly diluted into inositol-free medium and incubated for an additional 4–5 hours prior to fluorescence imaging or cycloheximide chase assays. In control experiments, 100 μM inositol was added to the inositol-free medium.

Fatty acid–supplemented growth media were prepared as previously described [41]. Briefly, standard SC medium was modified to contain 0.1% glucose and 1.5% Brij L23 (Sigma-Aldrich), and supplemented with 16 mM fatty acids (oleic acid or linoleic acid; Sigma-Aldrich). Control media were prepared identically but without the addition of fatty acids.

### Growth test

Yeast growth was assessed using a serial dilution spot assay. Yeast strains were cultured overnight in standard growth medium at 30°C with shaking until they reached mid-log phase. Cultures were then adjusted to an OD_600_ of 1.0, and a series of 5-fold serial dilutions were prepared in sterile water. Five microliters of each dilution were spotted onto appropriate solid agar plates using a multichannel pipette. Plates were incubated at the indicated temperatures and time points, and growth was documented by imaging. Representative images were taken after the appropriate incubation period to compare growth differences between strains.

### Fluorescence microscopy imaging

Fluorescence microscopy was performed using a Zeiss Axiovert 200M fluorescence microscope (Carl Zeiss MicroImaging) equipped with standard Zeiss filter sets for DAPI, FITC/GFP and TRITC/RFP. Yeast cells were grown to mid-log phase in the indicated media, harvested by centrifugation, and resuspended in phosphate-buffered saline (PBS) prior to imaging. Live-cell imaging was performed using a 100 × 1.3 NA oil immersion objective, and images were captured with a monochrome digital camera (Axiocam; Carl Zeiss MicroImaging). For each field, z-stacks spanning 5 µm were collected at 0.2 µm step intervals. Following image acquisition, the stacks were deconvolved and projected to generate 2D images for subsequent quantitation. Exposure times and imaging settings were kept constant across all samples to ensure comparability. Image acquisition, deconvolution, and analysis were carried out using Zeiss Zen software.

To quantify cortical ER (cER) inheritance, more than 100 budded cells were analyzed per condition. Cells were classified into three categories based on bud index (the ratio of bud size to mother cell diameter), and the presence or absence of cER in the bud was scored, as previously described [42].

Nuclear DNA was stained with DAPI to visualize nuclear position and facilitate identification of perinuclear ER localization in live cells. Cells were incubated with 1 µg/mL DAPI in PBS for 5–10 minutes at room temperature, followed by a PBS wash prior to imaging.

### IET tubule measurement protocols

To quantify initial ER tubules (IET)—a recently introduced term describing nascent ER tubules that emerge from the perinuclear ER and extend into the bud [43]—fluorescence microscopy images were analyzed using ImageJ software. Yeast cells expressing fluorescently tagged ER markers were grown to mid-log phase in appropriate media and imaged using a Zeiss fluorescence microscope equipped with a 100 × oil immersion objective. Images were acquired under identical exposure settings to ensure consistency across samples.

For image analysis, raw fluorescence images were opened in ImageJ, and background subtraction was performed using the “Subtract Background” function. Tubules were manually traced using the “Freehand Line” tool or the “Segmentation” tool for more precise edge detection. The “Measure” function was then used to determine tubule length in pixels, which was converted to micrometers based on the microscope’s calibration settings. At least 50 tubules per condition were measured from multiple fields of view. To quantify tubule fluorescence intensity, tubules were selected as regions of interest (ROIs) using the “Freehand Selection” or “Polygon Selection” tool, ensuring that the entire tubule was encompassed. The “Measure” function was then used to determine the mean fluorescence intensity and area of the ROI. Background fluorescence was subtracted by measuring an adjacent non-fluorescent region. At least 50 tubules per condition were analyzed across multiple fields of view. Statistical analysis was conducted using Microsoft Excel, with data analyzed using a student’s t-test. Results are presented as mean ± standard error of the mean (SEM).

### UPRE-GFP assay and quantitation

The unfolded protein response element (UPRE)-GFP reporter assay was used to assess UPR activation. Yeast cells carrying the UPRE-GFP reporter plasmid were grown to mid-log phase in selective media at 30°C. Cells were then either left untreated or subjected to ER stress by treatment with 1ug/ml tunicamycin for 90mins. Following treatment, cells were harvested by centrifugation, washed with phosphate-buffered saline (PBS), and resuspended in PBS for fluorescence microscopy imaging.

Fluorescence images were captured using a Zeiss fluorescence microscope with a 100 × oil immersion objective. To quantify GFP intensity, images were analyzed using ImageJ software. Regions of interest (ROIs) were manually selected to measure fluorescence intensity within individual cells. Background fluorescence was subtracted, and mean fluorescence intensity (MFI) was calculated for each condition. Data were normalized to cell count and analyzed for statistical significance using Excel t-test.

### Cycloheximide chase degradation assay

The degradation of epitope-tagged proteins was assessed using a cycloheximide chase assay as described [44]. Briefly, yeast cultures grown to the logarithmic phase were treated with 100 μg/ml cycloheximide to inhibit protein synthesis. Samples were then collected at various time points post-treatment, lysed, and subjected to immunoblotting to evaluate protein degradation, following the protocol outlined by [44]. GFP-tagged and HA-tagged proteins were detected using anti-GFP (Roche, catalogue number 11814460001) and anti-HA monoclonal antibodies (16B12, Covance, catalog number MMS-101P-500). Pgk1 is used as loading control and anti-Pgk1 is provided from Invitrogen (catalog number 459250).

## Results

### ER lipid saturation via OLE1 regulates cortical ER inheritance at late stages

**Fig 1:**
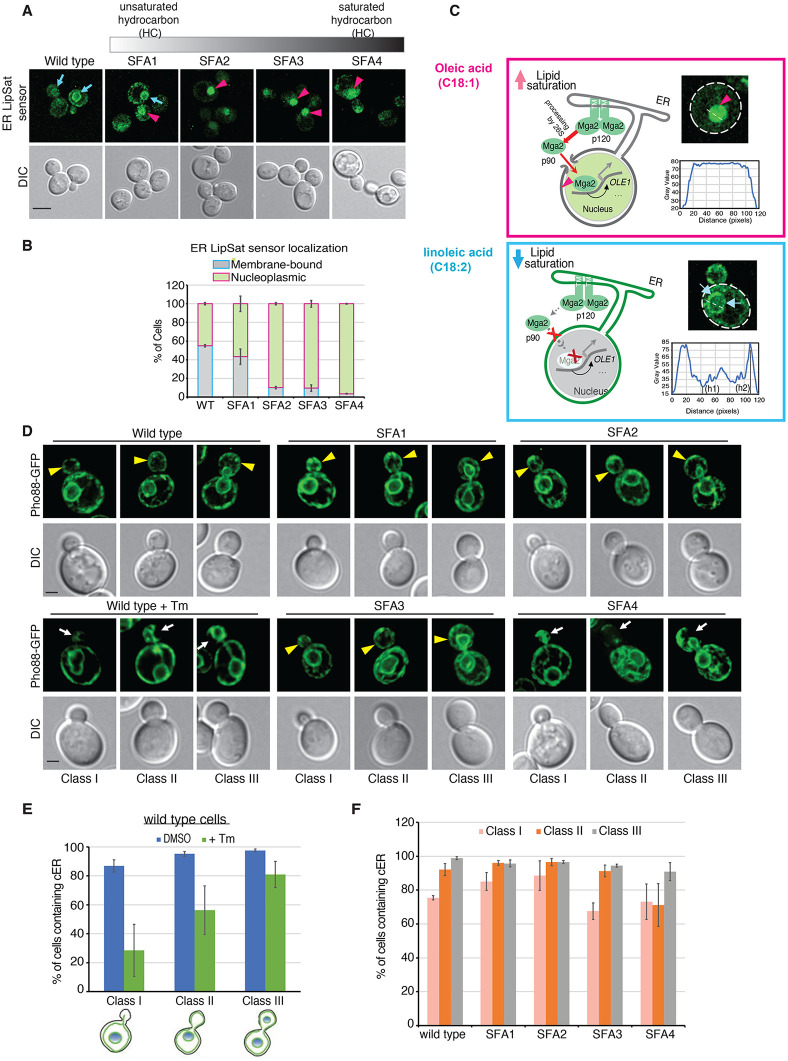
Lipid Saturation level has effects on ER inheritance. (**A–B**) Analysis of ER lipid saturation levels in SFA1–SFA4 strains. (**A**) Representative fluorescence images showing localization of the ER LipSat sensor in wild type (MNY3414, W303 background) and SFA1-SFA4 strains. Cells were grown in standard SC medium containing 2% glucose. A grayscale bar above the images illustrates gradual changes in levels of saturated fatty acids. Blue arrows mark membrane-associated ER LipSat sensor, while red arrowheads indicate nucleoplasmic localization. (**B**) Quantification of membrane-bound versus nucleoplasmic ER LipSat sensor localization. Fluorescence distribution was measured from >100 cells per strain. Scale bar, 5 μm. (**C**) The ER LipSat sensors enable assessment of fatty acid (FA) saturation in the ER membrane. Mga2, an ER membrane localized transcription factor, undergoes proteolytic cleavage when membrane saturation increases, allowing its N-terminal portion to translocate into the nucleus to activate transcription. Therefore, the relative distribution of nuclear versus ER-localized Mga2 serves as a readout for ER membrane saturation. Without elevated FA saturation, ER LipSat sensor stays on the ER Membrane. Diagram was adapted from Romanauska et al., 2021 [41]. (**D**) Cortical ER (cER) inheritance defects in SFA4. ER was visualized with a well-established ER marker Pho88-GFP. Representative Live-cell images of wild-type and SFA1–SFA4 strains expressing Pho88-GFP are shown. For comparison, wild-type cells treated with 1 μg/mL tunicamycin (Tm) for 90 min were included to induce ER stress. Yellow arrowheads indicate normal cER in daughter cells, while white arrows point to defective cER. Cells were classified into categories I–III as previously described [29]. Scale bar, 2 μm. (**E**)-(**F**) Quantification of the cER inheritance in class I, II and III for WT (**E**) and SFA1-SFA4 cells (**F**). Over 100 cells per class were analyzed, with data representing the average of three independent experiments. Error bars denote standard deviation (SD).

To investigate whether alterations in ER lipid saturation influence the unfolded protein response (UPR), the ER surveillance (ERSU) pathway, and ER-associated degradation (ERAD), we employed a previously established system in *Saccharomyces cerevisiae* that modulates lipid saturation through promoter variants controlling *OLE1* expression [36] (Figs 1A and S1A). *OLE1* encodes the sole fatty acid desaturase in yeast, introducing double bonds into saturated fatty acids (FA) which results in changes of the membrane fluidity. Gradual reduction of *OLE1* expression was achieved by promoter mutagenesis, generating four yeast strains (SFA1-SFA4) with progressively increased membrane phospholipid (PL) saturation and a decreasing phosphatidylethanolamine (PE)/phosphatidylcholine (PC) ratio. Mass spectrometry analysis from the previous study reported that SFA1 featured a PL composition similar to WT cells, which was followed by SFA2 and SFA3 with moderate increases, and finally, SFA4 displayed the most pronounced increase in saturated FA, which was characterized by a marked rise in fully saturated phospholipid species and shortened acyl chain length (decreasing from ~C16–C18 species toward shorter chains) [36]. In addition, the PE/PC ratio decreased across the SFA strains, reflecting broader remodeling of ER membrane lipid composition [36].

To visualize lipid saturation levels within different ER sub-regions, particularly the inner nuclear membrane (INM) and perinuclear ER (pnER), we applied Mga2-GFP–based lipid saturation (LipSat) reporters based on Mga2-GFP [41]. Mga2 is an ER-resident transcription factor whose GFP-tagged form localizes to both cortical ER (cER) and pnER [13] (S1B and S1C Fig). Under elevated lipid saturation, Mga2 undergoes proteolytic cleavage within its transmembrane domain, releasing a nuclear-targeted fragment that accumulates in the nucleoplasm [45,46]. Conversely, under reduced saturation, Mga2 remains membrane-bound without cleavage. Quantification of GFP signals in the nucleoplasm versus ER membranes (cER/pnER) thus provides a rapid readout of the relative distribution of saturated versus unsaturated lipids in ER membrane or INM. In wild-type control cells, ~ 25% of ER LipSat sensor GFP fluorescence localized to the membrane fraction, while ~75% appeared in the nucleus (S1D Fig). Supplementing cultures with oleic acid (C18:1), which moderately reduces lipid saturation, led to a slight decrease in Mga2 cleavage and nuclear GFP accumulation. Linoleic acid (C18:2), which more strongly reduces saturation, further decreased Mga2 cleavage and favored membrane localization of the sensor (S1D Fig). Comparable patterns were observed for the INM LipSat sensor GFP fluorescence (S1E Fig). These outcomes mirrored prior studies and validated the reporter’s performance in our system [41].

Applying this ER LipSat reporter to the SFA strains, we observed that SFA1 behaved similarly to wild type (~60% membrane bound, ~ 40% nucleoplasmic), while SFA2, SFA3, and particularly SFA4 showed progressively higher Mga2 nuclear signals, demonstrating elevated lipid saturation (Fig 1A and 1B). We next asked how these lipid property changes influenced cortical ER (cER) inheritance, a process we assayed using Pho88-GFP, an integrated ER reporter [29]. As previously described, ER inheritance proceeds in a sequential manner in budding yeast [17,47]: during early cell cycle stages (class I), an initial ER tubule (IET) extends from the pnER toward the bud neck, enters the daughter cell, and anchors at the polarisome at the bud tip. This is followed by lateral spreading along the plasma membrane to establish the cortical ER beneath the daughter cortex (class II–III cells). Consistent with earlier reports, tunicamycin-induced ER stress abolished cER inheritance in many class I cells (Fig 1C and 1D). When cells encounter stress much later (class III), cells completed division, but cER inheritance was blocked in the next cycle [29].

In the SFA background, cER inheritance defects correlated with the degree of lipid saturation (Fig 1C, and 1E). SFA1 and SFA2 resembled wild-type. In contrast, SFA3 class I cells, and SFA4 class I–II cells, displayed reduced cER inheritance, indicating that early stages of inheritance are particularly sensitive to increased lipid saturation. In SFA4, the reduction was more noticeable in class II cells, although still less severe than the inheritance blocks triggered by tunicamycin (Figs 1D, 1E, and S2A). SFA3 and SFA4 thus exhibited unique inheritance profiles, with SFA4 showing the clearest impairment, especially in class II daughters, distinguishing it from the relatively intact inheritance seen in WT, SFA1, and SFA2.

**Fig 2:**
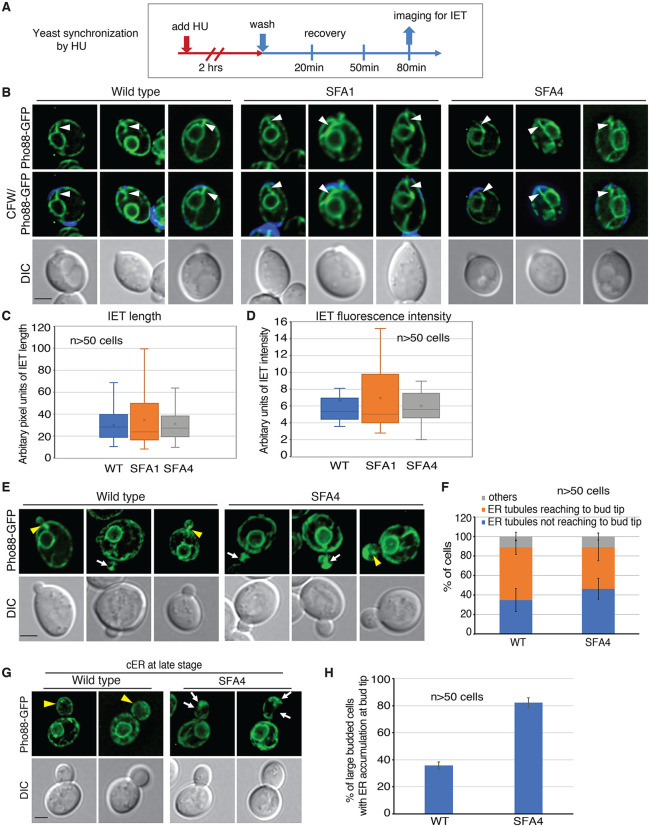
Effects of Lipid Saturation level on different stages of cortical ER (cER) inheritance. (**A**) Experimental diagram for hydroxyurea (HU) synchronization release into cell cycle. Yeast cells were synchronized in S-phase by treatment with 100 mM hydroxyurea (HU) for 2 hours. After HU treatment, cells were washed to remove HU and allowed to recover in fresh medium. Samples were collected at 20, 50, and 80 min post-wash to monitor progression through the cell cycle. Imaging for initial ER tubules (IET) was performed at 80 minutes after HU release. (**B**) The formation of initial ER tubules (IET) and their entry into the new bud in early stages of cER inheritance are not affected in SFA4 cells. Calcofluor White (CFW) staining marks cytokinetic remnants (CRMs). Three representative cells are shown for each indicated strain, with images from the GFP channel, merged GFP and DAPI channels, and DIC. Arrowheads indicate an IET extending from the perinuclear ER (pnER) in the mother cell toward the newly forming bud. Scale bar, 5 µm. (**C–D**) Quantification of IET tubule length and fluorescence intensity across different strains. Measurements were performed using ImageJ as described in Materials and Methods. Over 50 cells were analyzed per strain. (**E**) Representative images showing initial ER tubules (IET) extending into the bud at different stages of cER inheritance in wild-type and SFA4 cells. Yellow arrowheads indicate cells with an IET extending a half way into the daughter cell but not reach to the bud tip, while white arrows highlight IET extension reaching fully to bud tip. Scale bar, 5 µm. (**F**) Quantification of cells exhibiting different ER tubule positions. Depending on the inheritance stage, ER tubules either dynamically extend to the bud tip or fail to do so. Over 50 cells were analyzed per strain. (**G**) cER in large buds during the late stages of cell cycle does not extend all the way through the cortex of the daughter cell in SFA4 cells. Yellow arrowheads indicate normal cER distribution in wild-type cells, while white arrows highlight ER accumulation at the bud tip and a lack of cER around the bud cortex in SFA4 cells. Scale bar, 2 µm. (**H**) Quantification of cER accumulation at the bud tip in large-budded wild-type and SFA4 cells. Over 50 cells were analyzed per strain.

To investigate whether early steps of ER inheritance were disrupted in SFA4, we analyzed IET initiation and orientation in synchronized class I or II cells. Cells were arrested at G2/M with hydroxyurea and released into G1, capturing populations enriched for incipient bud formation (Fig 2A and 2B). Using calcofluor white (CFW) staining to visualize bud scars, we monitored IET orientation relative to the bud neck. Operationally, IETs were defined as tubular extensions emerging from the pnER toward the bud. Quantitative imaging revealed no significant differences between WT and SFA4 in IET frequency, timing, length, or intensity, nor in the proportion of tubules reaching into the bud (Fig 2B–2F). Thus, early ER targeting events remained largely intact in SFA4.

Striking defects emerged instead in class II cells. Compared to ~30% of WT daughters, nearly 80% of SFA4 class II buds accumulated ER markers abnormally at the bud tip, with little or no cortical ER along the bud cortex (Fig 2G and 2H). Although these inheritance defects were milder than those observed under acute ER stress, SFA4 uniquely exhibited stronger impairment in class II than in class I or III cells. Taken together, these findings indicate that excess ER lipid saturation does not disturb the earliest targeting events of ER inheritance but selectively disrupts later stages, particularly the maturation and distribution of cortical ER in class II daughter cells.

### Differential eeffects of ER lipid saturation on ERAD substrate degradation

**Fig 3:**
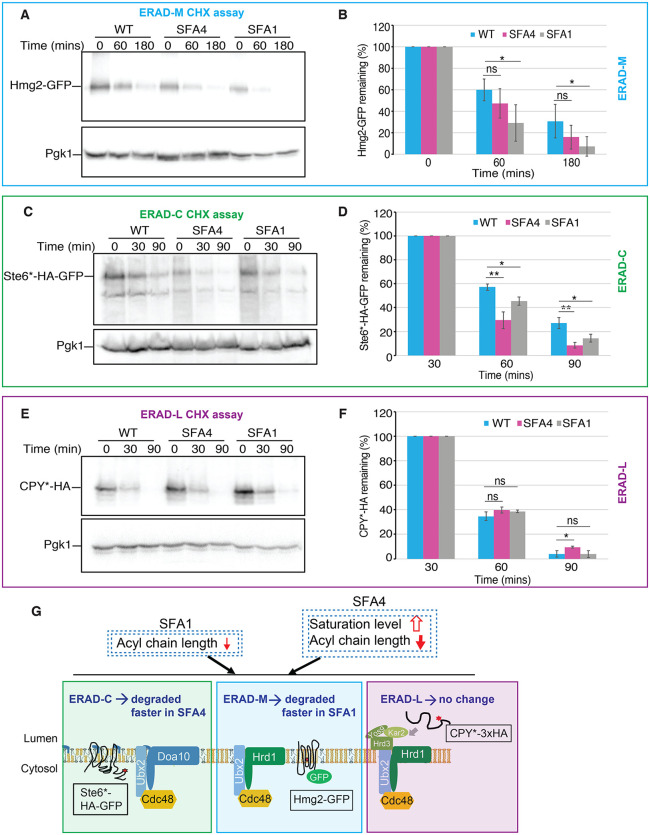
Effects of Lipid Saturation level on ERAD. (**A**)-(**E**) A cycloheximide (CHX) chase of Hmg2-GFP (A-B), CPY*-HA (**C** and **D**), and Ste6*-HA-GFP (**E** and **F**) expressed in Wild-type, lipid saturation mutants, SFA1 and SFA4. Upon CHX treatment, equal numbers of cells were collected to prepare total cell extracts for SDS-PAGE, followed by western blot analysis to detect ERAD membrane (ERAD-M) substrate Hmg2-GFP, ERAD luminal substrate (ERAD-L) CPY*-HA, and ERAD cytosolic substrate (ERAD-C), Ste6*-HA-GFP. Pgk1 was used as a loading control. The mean percentage of each ERAD substrate remaining in the cells for at least three biological replicates is plotted. Error bars represent the standard deviation (SD). A one-tailed *t-test* was used to determine the significance of the difference between WT and SFA4 cells or between WT and SFA1 cells. p < 0.05, *; *p < 0.005*, **; p < 0.001, ***. (**G**) Schematic summary of ERAD substrate degradation in SFA1 or SFA4 yeast strains. In SFA1 yeast cells, membrane lipids display wild-type like saturation with slightly shortened acyl chain length. In contrast, SFA4 cells exhibit both markedly reduced acyl chain length and increased saturation of lipid membrane. These changes in membrane composition selectively enhance the degradation of ERAD-C and ERAD-M substrates–Ste6*-HA-GFP and Hmg2-GFP, respectively– while having no effect on the degradation of the ERAD-L substrate CPY*-3xHA. ERAD-C and ERAD-M substrates are recognized by the Doa10 and Hrd1 ubiquitin ligase complexes, respectively, and extracted by the Cdc48-Ubx2 complex. ERAD-L substrate degradation depends on the Hrd1-Hrd3 complex and the ER luminal chaperones Kar2 and Yos9.

Recognizing the essential role of phospholipid homeostasis in ER function and cellular stress adaptation, we next explored how the saturation state of ER membrane lipids modulates the efficiency of ER-associated degradation (ERAD). Specifically, we quantified the half-lives of distinct ERAD substrates by monitoring their steady-state protein levels over time following cycloheximide (CHX)–mediated inhibition of protein synthesis—a well-established method to determine protein degradation dynamics (Fig 3). By arresting new protein synthesis with CHX, we could precisely track the decrease in substrate abundance as a direct readout of degradation, allowing robust determination of protein half-life and stability under varying lipid saturation conditions. Using this cycloheximide chase assay, we probed the fate of three well-established ERAD substrates: Hmg2-GFP—an integral membrane protein processed via the ERAD-M pathway (mutation in the membrane domain), CPY*-HA—a mutated soluble luminal protein (G255R in carboxypeptidase Y) cleared by the ERAD-L pathway (luminal domain misfolding), and Ste6–166-3xHA-GFP (Ste6*-HA-GFP)—a mutant membrane protein with a misfolded cytosolic domain, handled by the ERAD-C pathway (cytosolic domain misfolding) (Fig 3A–3F). Under these conditions, Hmg2-GFP degradation was modestly but reproducibly accelerated in both SFA1 and SFA4 mutants compared with wild-type cells, with the effect more pronounced in SFA1 and less evident in SFA4 (Fig 3A and 3B). The degradation of Ste6*-HA-GFP was also enhanced in both mutants but was most pronounced in SFA4 (Fig 3C and 3D). In contrast, the clearance of CPY*-HA remained essentially unchanged across wild-type, SFA1, and SFA4 backgrounds (Fig 3E and 3F).

These results reveal a specificity: membrane lipid saturation may selectively accelerate the turnover of ERAD substrates with transmembrane or cytosolic misfolded domains, while leaving the degradation rate of soluble luminal proteins largely unaltered. Notably, SFA1 cells—enriched in unsaturated lipids— had a particularly strong impact on the stability of Hmg2-GFP, whereas SFA4 cells—with increased membrane saturation—most significantly reduced the half-life of Ste6*-HA-GFP (Fig 3G). Together, our findings point to potentially distinct and substrate-specific influences of ER membrane lipid saturation on ERAD efficiency, suggesting the lipid saturation change might act as a modulator of protein quality control within the ER.

### Lipid saturation levels of the ER membrane drive distinct UPR activation dynamics

**Fig 4:**
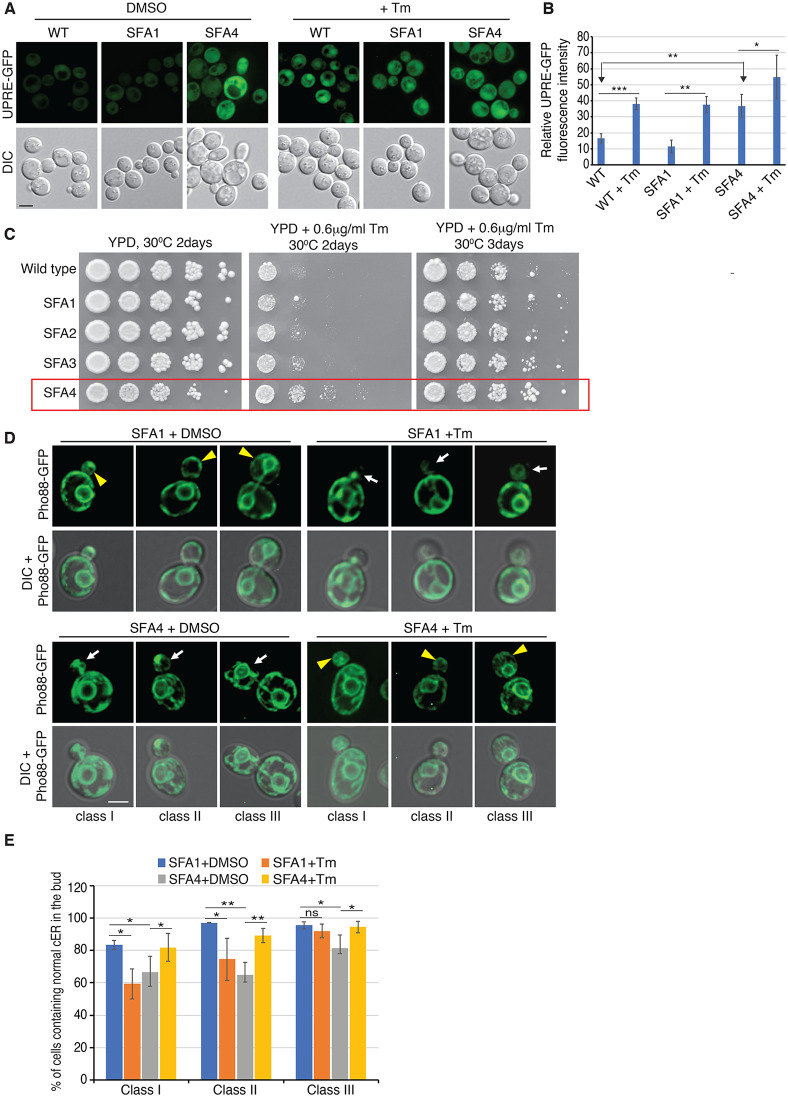
Effects of lipid saturation level on UPR pathways. (**A**) Expression of GFP in yeast cells carrying the UPRE-GFP reporter, where the unfolded protein response element (UPRE) was fused to GFP and expression of GFP reports levels of UPR activation by ER stress. Wild-type (WT), SFA1 or SFA4 yeast cells harboring the UPRE-GFP plasmid were incubated with control DMSO or 1 µg/ml Tm for 90 minutes for examining the ER stress activation levels. Representative images for each condition are shown. Scale bar, 5 µm. (**B**) Quantitating over 300 cells carrying UPRE-GFP, in either WT, SFA1 or SFA4 cells, with or without Tm treatment. GFP expression in SFA4 cells even prior to Tm treatment shows that SFA4 cells induce ER stress and activate UPR. Mean fluorescence intensity with standard error of the mean is shown. A one-tailed *t-test* was used to determine the significance of the difference between WT and SFA4 cells or between SFA4 cells with and without Tm treatment. p < 0.05, *; *p < 0.01*, **; p < 0.002, ***. (**C**) Growth phenotypes of SFA1 - SFA4 yeast strains with or without Tm. Serial dilutions of overnight cultures from the indicated strains were plated on YPD plates or YPD plates containing 0.6 µg/ml Tm and grown at 30°C for 2–3 days. The red box highlights the improved growth of the SFA4 strain on YPD plates containing 0.6 µg/ml Tm. (**D**) cER inheritance in SFA1 and SFA4 cells upon treatment with Tm, an ER stress inducer. SFA1 or SFA4 strains expressing Pho88-GFP were treated with control DMSO or Tm for 90 minutes and then examined by fluorescence microscopy. Representative images of GFP or overlay of DIC with GFP of class I, II, and III cells are shown. Yellow arrowheads indicate normal cER in the bud, and white arrows point to the discontinuous cER under the cortex of the daughter cell. Scale bars, 5 µm. (**E**) Quantification of the cER inheritance of the cell shown in (D). For each condition, over 100 cells were analyzed. Results represent analyses from three independent experiments. Error bars denote standard deviation (SD). A one-tailed *t-test* was used to determine the significance of the difference between SFA1 and SFA4 cells with and without Tm treatment, p < 0.01*,* **; p < 0.05, *; ns, not significant.

To determine whether increased ER membrane lipid saturation influences UPR activity, we monitored UPR induction using a UPRE-GFP reporter in wild-type (WT), SFA1, and SFA4 yeast cells. Cells were transformed with a well-established reporter plasmid expressing GFP under the control of a UPR transcriptional element (UPRE), and GFP fluorescence was quantified using ImageJ. As expected, treatment with tunicamycin (Tm; 1 µg/mL for 1.5 hours) led to an approximately two-fold increase in GFP fluorescence in WT cells, consistent with previous findings [22]. SFA1 cells treated with Tm exhibited GFP levels similar to those of Tm-treated WT cells (Fig 4A and 4B).

Remarkably, SFA4 cells displayed elevated GFP fluorescence even without Tm treatment, reaching levels comparable to those observed in Tm-treated WT or SFA1 cells. This strong basal activation indicates that increased ER membrane lipid saturation in SFA4 is sufficient to induce UPR activation independently of external ER stressors. Upon Tm treatment, GFP levels in SFA4 cells increased further, demonstrating that the UPR machinery remains responsive to additional ER stress (Fig 4A and 4B). These results support the idea that ER lipid saturation perturbs ER homeostasis and activates the UPR independently of unfolded protein accumulation, likely through direct sensing of membrane lipid composition by UPR transducers Ire1 in yeast. To corroborate the findings from the UPRE-GFP reporter assay, we evaluated the growth phenotypes of WT and SFA strains under ER stress conditions (Fig 4C). Under unstressed conditions, SFA1, SFA2, and SFA3 strains exhibited growth comparable to WT. In contrast, SFA4 cells showed slower growth, consistent with a basal stress state. Upon ER stress induction with tunicamycin (Tm), all strains except SFA4 displayed a growth defect. Interestingly, SFA4 cells exhibited improved growth in the presence of Tm relative to their untreated condition, and even outperformed other SFA strains under ER stress. These results suggest that Tm-induced proteotoxic stress activates the UPR in a way that may partially compensate for the lipotoxic defects in SFA4 cells.

To further investigate the unexpected behavior of SFA4, we investigated if/how ER stress alters the cortical ER (cER) inheritance when compared to that observed in unstressed SFA4 cells. In all classes of cells for SFA4, induction of ER stress with Tm restored cER inheritance, enabling all cells to properly inherit cER despite significant alterations in overall ER morphology (Fig 4D, 4E, and S2B). Notably, unstressed SFA4 class II cells exhibited a pronounced decrease in cER inheritance (Fig 4E, class II, gray bar). However, upon induction of ER stress with Tm, the reduced cER inheritance in SFA4 class II cells was restored to nearly normal levels (Figs 4D, 4E, and S2B). This was in contrast to SFA1 cells; when we assessed cER inheritance across all three classes with ER stress, cER inheritance was blocked in class I, II, and III buds in a manner similar to that observed in wild-type cells (Fig 4D and 4E). These findings suggest that elevated lipid saturation in SFA4 impacts ER inheritance efficiency, while ER stress-induced changes in lipid composition could facilitate improved cER inheritance. Alternatively, ER stress may alter the biophysical characteristics of yeast ER lipids in a way that enhances the effectiveness of cER inheritance.

### Impact of inositol depletion on cortical ER inheritance and ERAD substrate degradation

**Fig 5:**
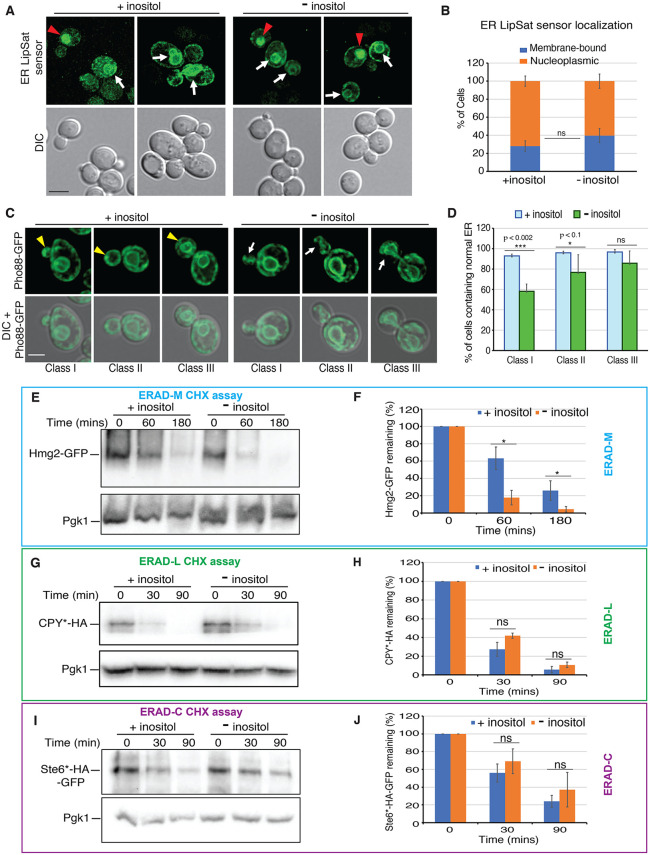
Inositol depletion affects cER inheritance and ERAD substrate degradation. (**A**) ER membrane lipid saturation levels in response to inositol depletion. Live cell imaging of wild-type cells expressing the plasmid-based ER LipSat sensor under conditions with or without 100 µM inositol supplementation. Red arrowheads indicate nucleoplasmic localization, and white arrows point to nuclear envelope localization. Scale bar, 5 µm. (**B**) Localization of the ER LipSat sensor was quantitated to assess lipid saturation levels. Sensor distribution was classified into two phenotypes: membrane-bound (indicative of lipid unsaturation level) or nucleoplasmic (indicative of lipid saturation level). Data are presented as mean values and standard deviations. A one-tailed *t-test* shows no significant difference (ns, p > 0.1) for ER lipid saturation between the two growth conditions. (**C**) Inositol depletion leads to defects in cER inheritance. Wild-type cells expressing the ER membrane reporter, Pho88-GFP, were analyzed by fluorescence microscopy under normal or inositol-depleted conditions. Representative images show abnormal cER inheritance, with yellow arrowheads indicating normal cER formation in the bud and white arrows indicating defective cER. Scale bar, 5 µm. (**D**) Quantification of cER inheritance defects. More than 100 cells were analyzed under conditions of inositol supplementation (+Inositol) or depletion (-Inositol). Data represent the average of three independent experiments, with error bars indicating standard deviation (SD). Statistical significance of cER inheritance differences in wild-type cells grown with or without inositol was assessed using a one-tailed *t-test*. p < 0.002, ***; p < 0.1*,* *; ns, not significant. (**E**) Cycloheximide (CHX) chase degradation assay was performed for ERAD-M substrate, Hmg2-GFP, and analyzed by quantitating remaining Hmg2-GFP at each time point following the release from cycloheximide chase. (**F**) Quantification of ERAD-M (Hmg2-GFP) levels shows the percent of Hmg2-GFP remaining at each time point from the cycloheximide chase assay in panel (E). (**G**)-(**H**) Cycloheximide chase degradation assay was performed for ERAD-L substrate, CPY*-3xHA and analyzed by quantitating remaining CPY*-3xHA at each time point following the release from cycloheximide chase. (**I**)-(**J**) Cycloheximide chase degradation assay for ERAD-C substrate, Ste6*-HA-GFP, In all cases (E-H), Pkg1 was used for loading controls for all the quantitation. In all cases, means of percent remaining for each ERAD substrate from at least three biological replicates are plotted, with error bars representing standard deviation (SD). A one-tailed *t-test* was used to determine the significance of the difference between WT cells grown with and without inositol. p < 0.05, *; *p < 0.005*, **; p < 0.001, ***.

Outcomes of above studies have motivated us to test other types of lipid changes on ER inheritance. Inositol is a vital precursor for the synthesis of phosphatidylinositol (PI) in yeast. And lipid saturation levels modulate how inositol or inositol-containing lipids are incorporated and/or function in membranes [48]. Furthermore, previous research has demonstrated that inositol depletion activates the unfolded protein response (UPR) and leads to significant alterations in cellular lipid composition [49,50]. To investigate whether membrane alterations caused by inositol depletion affect ER quality control, we employed LipSat sensors (S1B and S1C Fig) to assess ER or INM membrane lipid saturation under inositol-depleted conditions (Fig 5A and 5B and S3A-S3B). Our analysis revealed that inositol deprivation does not cause major changes to lipid saturation levels at the ER or INM, suggesting that the observed effects on the ER homeostasis are not due to altered membrane saturation.

We next examined the impact of inositol depletion on cER inheritance and ERAD pathways. Under inositol-depleted conditions, early-stage (Class I) cells successfully formed ER tubules emerging from the pnER, extending into the bud and anchoring at the bud tip. However, in later-stage (Class II) cells, while ER tubules persisted, cER formation was partially inhibited or delayed. By the final stage (Class III), despite proper pnER being established in the bud, cER failed to uniformly distribute along the bud cortex (Figs 5C and S3C). Quantitative analysis confirmed significant defects in cER inheritance due to inositol depletion, although these defects were less severe than those induced by tunicamycin (Tm)–mediated ER stress (Fig 5D**,** in comparison to Fig 1D).

To assess the influence of inositol depletion on ERAD, we measured the turnover of three distinct ERAD substrates. Under inositol-depleted conditions, degradation of the ERAD-M substrate Hmg2-GFP accelerated (Fig 5E and 5F). In contrast, both the ERAD-L substrate CPY*-HA (Fig 5G and 5H) and the ERAD-C substrate Ste6*-HA-GFP (Fig 5I and 5J) exhibited mild stabilization, with only a slight decrease in their degradation rates, suggesting a modest impairment in the breakdown of these substrates. Previous studies have reported that inositol depletion alters ER membrane phospholipid composition—specifically decreasing PI levels while elevating cardiolipin (CL) and phosphatidylglycerol (PG) levels [51–53] (S4A Fig). These changes modulate overall membrane lipid homeostasis, which collectively enhances the degradation of ERAD-M substrates. In contrast, the degradation of ERAD-L and ERAD-C substrates remains largely unaffected, indicating that lipid perturbation under inositol depletion selectively impacts the ERAD-M pathway. To determine whether inositol depletion–induced membrane aberrations act synergistically with increased ER membrane lipid saturation, we assessed the growth of wild-type and SFA1–SFA4 strains on medium with or without inositol. Wild-type, SFA1, and SFA2 cells displayed no growth defects under inositol deprivation. However, the SFA3 strain grew slightly slower at 3^0°^C under inositol-depleted growth condition and somewhat increased sensitivity at elevated temperature of 37^°^C (S4B Fig, blue box). This trend became more pronounced for SFA4, where growth at 30°C on -inositol plates was reduced markedly (S4B Fig, red box). These results indicate a synthetic interaction between inositol depletion and elevated ER membrane lipid saturation.

## Discussion

The ER is a highly dynamic organelle, playing a central role in protein folding, lipid synthesis, and being faithfully inherited during division. Our findings suggest that ER lipid composition—including saturation level, acyl chain length, and phospholipid headgroup identity—can influence ER architecture, dynamics, and proteostasis. Disruptions to lipid balance, such as inositol depletion or increased lipid saturation, impair ER inheritance in specific ways and differentially affect branches of ERAD.

Lipid saturation, in particular, emerged as a central determinant of ER membrane biophysical properties and function [35,54]. Our results have shown that in SFA4 cells, which accumulate more saturated lipids, membranes may be less flexible, potentially hindering cortical ER (cER) inheritance and causing structural defects during polarized growth. By contrast, SFA1 cells, which maintain shorter or more unsaturated acyl chains, exhibited relatively mild effects, suggesting that increased membrane flexibility may help buffer the impact of lipid perturbations.

Under ER stress conditions, ER inheritance to the daughter cell is blocked by the ER stress surveillance (ERSU) pathway, which is mediated by Rtn1, Yop1, and the MAP kinase Slt2 [29,55]. Previous work in our lab demonstrated that the ER stress–induced sphingolipid phytosphingosine (PHS), is specifically recognized by Rtn1 and Yop1 through their PHS-binding motifs [43,56]. This recognition triggers activation of Slt2, culminating in the blockade of cER inheritance. Building on these insights, we and others have shown that the daughter cell’s cER is seeded by the initial ER tubule (IET) that emerges from the mother’s perinuclear ER (pnER), extends through the bud neck, and ultimately reaches the bud tip [47,57]. Under ER proteotoxic stress, this IET is redirected away from the bud neck and instead targets the most recent bud scar [18]. Mutations in Rtn1 or Yop1 that disrupt PHS binding abrogate the cell’s ability to reroute the IET to the bud scar, leading to a failure in ER inheritance blockade—much like the phenotype seen in slt2, rtn1, or yop1 knockout cells. These findings emphasize the necessity of ERSU in preventing a stressed ER from entering the daughter cell [18] and are consistent with a role for lipid composition in modulating ER inheritance, although the precise contributions of lipid saturation require further investigation.

Our current findings suggest that lipid saturation influences different stages of cortical ER (cER) inheritance. Previously, we showed that under ER proteotoxic stress, cER inheritance is most vulnerable at an early stage, defining a “point of no return” that blocks ER transmission to the daughter cell [47,56]. Specifically, when cells experience ER proteotoxic stress, the inheritance block occurs early—before the initial ER tubule (IET) emerging from the peri-nuclear ER (pnER) enters the daughter cell, remaining trapped in the mother cell. However, the cER inheritance block triggered by increased lipid saturation, as in SFA4 cells, appears to act at a later stage. In class I cells bearing small buds, the IET successfully enters the daughter cell and reaches the bud tip, but fails to expand beneath the daughter cortex. This suggests that elevated lipid saturation heightens sensitivity at a later inheritance step, likely by altering membrane biophysics such as lipid packing and flexibility. Consistent with this, our previous findings implicate ICE2 and SLT2 in facilitating cER expansion along the daughter cortex, a process likely regulated in response to biophysical changes in ER membrane properties. Increased lipid saturation likely causes the ER membrane to become more tightly packed, hindering cER extension along the plasma membrane cortex. Thus, lipid composition critically determines the spatial stage at which the ER inheritance block occurs. Together, these data reveal an alternative, lipid-sensitive checkpoint that regulates cER inheritance during its cortical expansion phase in the daughter cell. Future studies will be essential to dissect the molecular pathways governing this distinct, lipid-dependent inheritance checkpoint and its integration with cellular stress responses.

Altered membrane composition shapes the efficiency of ER quality control [58,59]. Membrane lipid saturation selectively accelerates the turnover of ERAD substrates with transmembrane or cytosolic misfolded domains, while leaving the degradation of soluble luminal proteins largely unaltered. For example, SFA1 cells—enriched in unsaturated lipids—had a particularly strong impact on the stability of Hmg2-GFP, whereas SFA4 cells—with increased membrane saturation—most significantly reduced the half-life of Ste6*-HA-GFP. However, the acceleration of Hmg2-GFP or Ste6*-HA-GFP degradation does not strictly correlate with the overall degree of membrane lipid saturation, suggesting that local membrane microenvironments or specific lipid–protein interactions, rather than global bilayer composition, are critical determinants of ERAD substrate recognition and processing.

Saturated membrane may enhance degradation of certain ERAD substrates by making misfolded proteins less stable within a more tightly packed lipid bilayer, thereby affecting recognition and processing. This supports models in which the biophysical properties of the lipid bilayer modulate substrate conformations and their accessibility to degradation machinery. Yet, ERAD-L substrates such as CPY*-HA are largely spared, highlighting differences in the sensitivity of ERAD branches to changes in membrane composition. Indeed, the divergent properties of the SFA strains offer mechanistic insight. SFA1 cells maintain a largely wild-type phospholipid profile with shorter chains, whereas SFA4 cells accumulate fully saturated phospholipids that generate rigid membranes [36]. In wild-type cells such saturated lipids are present only as part of a more diverse lipid pool, buffering their impact. As summarized in Fig 3G, these compositional differences likely exert substrate-specific effects, influencing recognition, ubiquitination, or retrotranslocation of membrane-associated ERAD substrates such as Hmg2-GFP and Ste6*-HA-GFP [31,33]. Each of these steps depends on dynamic interactions between misfolded domains and membrane-embedded quality control factors, processes that are highly sensitive to bilayer rigidity. In contrast, CPY*-HA, a soluble luminal substrate requiring recognition by chaperones such as BiP, bypasses reliance on the membrane environment and is therefore less directly affected by lipid saturation, although ERAD-L substrates require to pass through the lipid bilayer to reach to the proteasomes present in cytoplasm.

Together, these findings reveal that ER lipid composition exerts a profound influence on ER inheritance and aspects of protein quality control. Lipid saturation and headgroup balance may influence membrane properties, which in turn determine how efficiently different ERAD branches recognize and process misfolded clients. The results underscore that proteostasis cannot be viewed independently of lipid metabolism; instead, membrane composition acts as an active regulator of quality control pathways, tailoring ER architecture and function to the metabolic state of the cell.

## Supporting information

S1 Fig Extent of Lipid saturation is measured by ER LipSat and INM LipSat sensors. (A) Schematic illustrating the modulation of Ole1 expression levels in SFA1–SFA4 yeast strains [36]. OLE1 expression is placed under the control of different promoters to progressively reduce desaturase activity and elevate membrane lipid saturation. Drawing was modified from Venkatraman et al., 2023 [36]. (**B**)(**C**) The ER LipSat (B) and INM LipSat (C) sensors [41] enable assessment of fatty acid (FA) saturation in the ER membrane and the inner nuclear membrane (INM). (B) Mga2, an ER membrane localized transcription factor, undergoes proteolytic cleavage when membrane saturation increases, allowing its N-terminal portion to translocate into the nucleus to activate transcription. Therefore, the relative distribution of nuclear versus ER-localized Mga2 serves as a readout for ER membrane saturation. (**C**) Similarly, the INM LipSat sensor reports on saturation at the INM (or perinuclear ER). Mga2 localized to the INM by the Heh2-Nuclear localization signal (NLS) undergoes cleavage in response to elevated FA saturation, with its N-terminal GFP-tagged fragment released into the nucleoplasm. Diagram was adapted from Romanauska et al., 2021 [41]. Without elevated FA saturation, INM LipSat stays on the INM. For each case, line plots showing GFP levels and localization of ER LipSat and INM LipSat sensors. GFP fluorescence was quantified along a line drawn across the center of the nucleus. The quantification method was adapted from Romanauska et al., 2021 [41]. Fluorescence intensity profiles with a single central peak were classified as nucleoplasmic. For profiles with two or more prominent peaks, the lowest valley intensity (h1) and highest peak intensity (h2) were measured, and the ratio h1:h2 was calculated. A ratio > 0.5 was classified as nucleoplasmic, whereas a ratio < 0.5 indicated membrane associated localization. Wild-type cells (BY strain background) expressing plasmid-based ER LipSat sensor (MNY3371) or INM LipSat sensor (MNY3372) were grown in special medium with 0.1% glucose plus 1.5% Brij L23 solution supplemented with 16mM of the indicated fatty acid. Yellow arrowheads indicate membrane-bound sensor localization, and white arrows mark nucleoplasmic localization. Scale bar, 5 μm. (**D**) and (**E**) Quantification of ER LipSat and INM LipSat sensor localization from (B) and (C). Cells were categorized based on sensor localization as membrane-bound or nucleoplasmic. Over 100 cells per strain were analyzed.
(JPG)

S2 Fig Effects of Lipid Saturation level on cER inheritance with or without ER stressor Tm treatment. (**A**) Fields of cells used for quantitation of cER inheritance in wild-type and SFA1–SFA4 cells shown in Fig 1D and 1E. Representative images of wild-type and SFA1–SFA4 cells expressing the ER membrane marker Pho88-GFP. Two different fields per specific strain are shown. Arrowheads indicate normal cER in daughter cells, while arrows mark examples of defective cER. Scale bar, 5 μm. (**B**) Tunicamycin (Tm) disrupts cER inheritance in SFA1 cells but rescues cER inheritance defects in SFA4 cells. SFA1 and SFA4 cells expressing Pho88-GFP were treated with 1 μg/ml Tm or DMSO (control) for 90 min before imaging. Two representative fields are shown. Arrowheads indicate examples of normal cER in daughter cells, while arrows mark defective cER. Scale bar, 5 μm.
(JPG)

S3 Fig Effects of inositol depletion on the extent of FA saturation by localization of INM LipSat sensor and cER inheritance. (**A**) INM lipid saturation levels in response to inositol depletion. Live-cell imaging of wild-type cells expressing the plasmid-based INM LipSat sensor under conditions with or without 100 µM inositol supplementation. Arrowheads indicate FA saturation of the INM as INM LipSat sensor localized to nucleoplasmic localization, while arrows point to membrane-bound localization. Scale bar, 5 µm. (**B**) Quantification of INM LipSat sensor localization in panel (A). Sensor localization was classified as either membrane-bound or nucleoplasmic. Data represent mean values ± standard deviation (SD). A one-tailed *t-test* shows no significant difference (ns, p > 0.1) for INM lipid saturation between the two growth conditions. (**C**) Inositol depletion leads to defects in cER inheritance. Wild-type cells expressing the ER membrane marker Pho88-GFP were examined by fluorescence microscopy under normal or inositol-depleted conditions. Representative fields are shown. Arrowheads indicate normal cER inheritance in the bud, while arrows highlight defective cER. Scale bar, 5 µm.
(JPG)

S4 Fig Synthetic growth phenotype of inositol depletion with membrane lipid saturation mutants SFA1 or SFA4 strains. (A) Cartoon summary illustrating the impact of inositol depletion on ER-associated degradation (ERAD) pathways. Under inositol-depleted conditions, degradation of ERAD-M substrates (e.g., Hmg2-GFP) is accelerated, while ERAD-C substrate (e.g., Ste6*-HA-GFP) degradation or ERAD-L substrate degradation (e.g., CPY*-3xHA) is not significantly affected. Substrate-specific E3 ubiquitin ligases and associated components are indicated: Hrd1 and Hrd3 for ERAD-L, Doa10 for ERAD-C, and Hrd1 for ERAD-M. Cdc48 and Ubx2 are involved in retrotranslocation and extraction for multiple ERAD branches. PI, PG and CL denote altered lipid environments resulting from inositol depletion, which may underlie differential effects of membrane alteration on substrate processing. (**B**) Growth phenotypes of SFA1 to SFA4 yeast in inositol-depleted conditions. Serial dilutions of overnight cultures from the indicated strains were plated on minimal synthetic plates with or without 100 µg/ml inositol and grown for 2 days at 30°C or 37°C. The blue and red boxes highlight the impaired growth of SFA3 and SFA4 strains under inositol depletion.
(JPG)

S1 File Supporting information for figures (“S1_raw_images 072426.jpg”). (JPG)

S1 Table Yeast strains used in this study. All yeast strains used in this study are listed. (DOCX)

S2 Table Plasmids used in this study. All plasmids used in this study are listed here. (DOCX)
