# Supplementary material for: Decoding the impact of lipid saturation on ER signaling networks, ERSU, UPR, and ERAD
Source: PLoS One. 2026 Aug 14;21(8):e0345910. doi: 10.1371/journal.pone.0345910 (PMC13475824; doi:10.1371/journal.pone.0345910)
Supplement: Supplementary file 1 [file pone.0345910.s006.docx]

**Table S1: Yeast strains used in this study.**

| **Strain**  **number** | **Relevant Genotype** | **Source** |
| --- | --- | --- |
| MNY3405 | *MATa*, *leu2-3,112, trp1-1, can1-100, ura3-1, ade2-1, his3-11,15* | Itay Budin lab |
| MNY3406 | *MATa, trp1-1, can1-100, ura3-1, ade2-1, his3-11,15, leu2-3,112::SFA1-Ole1* | Itay Budin lab |
| MNY3407 | *MATa, trp1-1, can1-100, ura3-1, ade2-1, his3-11,15, leu2-3,112::SFA2-Ole1* | Itay Budin lab |
| MNY3408 | *MATa, trp1-1, can1-100, ura3-1, ade2-1, his3-11,15, leu2-3,112::SFA3-Ole1* | Itay Budin lab |
| MNY3409 | *MATa, trp1-1, can1-100, ura3-1, ade2-1, his3-11,15, leu2-3,112::SFA4-Ole1* | Itay Budin lab |
| MNY3413 | *MATa*, *leu2-3,112, trp1-1, can1-100, ura3-1, ade2-1, his3-11,15::Pho88-GFP* | This study |
| MNY3424 | *MATa, trp1-1, can1-100, ura3-1, ade2-1, his3-11,15::Pho88-GFP, leu2-3,112::SFA1-Ole1* | This study |
| MNY3411 | *MATa, trp1-1, can1-100, ura3-1, ade2-1, his3-11,15::Pho88-GFP, leu2-3,112::SFA2-Ole1* | This study |
| MNY3410 | *MATa, trp1-1, can1-100, ura3-1, ade2-1, his3-11,15::Pho88-GFP, leu2-3,112::SFA3-Ole1* | This study |
| MNY3412 | *MATa, trp1-1, can1-100, ura3-1, ade2-1, his3-11,15::Pho88-GFP, leu2-3,112::SFA4-Ole1* | This study |
| MNY3414 | *MATa*, *leu2-3,112, trp1-1, can1-100, ura3-1, ade2-1, his3-11,15 [pRS313-pADH1-mGFP-mga2(128-1113)]* | This study |
| MNY3415 | *MATa, trp1-1, can1-100, ura3-1, ade2-1, his3-11,15, leu2-3,112::SFA1-Ole1 [pRS313-pADH1-mGFP-mga2(128-1113)]* | This study |
| MNY3416 | *MATa, trp1-1, can1-100, ura3-1, ade2-1, his3-11,15, leu2-3,112::SFA2-Ole1 [pRS313-pADH1-mGFP-mga2(128-1113)]* | This study |
| MNY3417 | *MATa, trp1-1, can1-100, ura3-1, ade2-1, his3-11,15, leu2-3,112::SFA3-Ole1 [pRS313-pADH1-mGFP-mga2(128-1113)]* | This study |
| MNY3418 | *MATa, trp1-1, can1-100, ura3-1, ade2-1, his3-11,15, leu2-3,112::SFA4-Ole1 [pRS313-pADH1-mGFP-mga2(128-1113)]* | This study |
| MNY3419 | *MATa*, *leu2-3,112, trp1-1, can1-100, ura3-1, ade2-1, his3-11,15 [pRS313-pADH1-mGFP-heh2(93-317)-mga2(128-1113)]* | This study |
| MNY3420 | *MATa, trp1-1, can1-100, ura3-1, ade2-1, his3-11,15, leu2-3,112::SFA1-Ole1 [pRS313-pADH1-mGFP-heh2(93-317)-mga2(128-1113)]* | This study |
| MNY3421 | *MATa, trp1-1, can1-100, ura3-1, ade2-1, his3-11,15, leu2-3,112::SFA2-Ole1 [pRS313-pADH1-mGFP-heh2(93-317)-mga2(128-1113)]* | This study |
| MNY3422 | *MATa, trp1-1, can1-100, ura3-1, ade2-1, his3-11,15, leu2-3,112::SFA3-Ole1 [pRS313-pADH1-mGFP-heh2(93-317)-mga2(128-1113)]* | This study |
| MNY3423 | *MATa, trp1-1, can1-100, ura3-1, ade2-1, his3-11,15, leu2-3,112::SFA4-Ole1 [pRS313-pADH1-mGFP-heh2(93-317)-mga2(128-1113)]* | This study |
| MNY3487 | *MATa*, *leu2-3,112, trp1-1, can1-100, ura3-1, ade2-1, his3-11,15 ire1Δ::KanMX* | This study |
| MNY2671 | *MATa his3Δ1 leu2Δ0 met15Δ0 ura3Δ0 slt2Δ::NATMX* | Lab collection |
| MNY3471 | *MATa*, *leu2-3,112, trp1-1, can1-100, ade2-1, his3-11,15, ura3-1::YIplac211-pTDH3-Hmg2-GFP* | This study |
| MNY3462 | *MATa, trp1-1, can1-100, ade2-1, his3-11,15, leu2-3,112::SFA1-Ole1 ura3-1::YIplac211-pTDH3-Hmg2-GFP* | This study |
| MNY3463 | *MATa, trp1-1, can1-100, ade2-1, his3-11,15, leu2-3,112::SFA4-Ole1 ura3-1::YIplac211-pTDH3-Hmg2-GFP* | This study |
| MNY3465 | *MATa*, *leu2-3,112, trp1-1, can1-100, ura3-1, ade2-1, his3-11,15 [pTDH3-Ste6-166-HA-GFP, 2u plasmid, URA3]* | This study |
| MNY3512 | *MATa, trp1-1, can1-100, ura3-1, ade2-1, his3-11,15, leu2-3,112::SFA1-Ole1 [pTDH3-Ste6-166-HA-GFP, 2u plasmid, URA3]* | This study |
| MNY3513 | *MATa, trp1-1, can1-100, ura3-1, ade2-1, his3-11,15, leu2-3,112::SFA4-Ole1 [pTDH3-Ste6-166-HA-GFP, 2u plasmid, URA3]* | This study |
| MNY3492 | *MATa, leu2-3,112, trp1-1, can1-100, ura3-1, ade2-1, his3-11,15 [YCp-CPY*-HA, Cen plasmid, URA3]* | This study |
| MNY3493 | *MATa, trp1-1, can1-100, ura3-1, ade2-1, his3-11,15, leu2-3,112::SFA1-Ole1 [YCp-CPY*-HA, Cen plasmid, URA3]* | This study |
| MNY3494 | *MATa, trp1-1, can1-100, ura3-1, ade2-1, his3-11,15, leu2-3,112::SFA4-Ole1 [YCp-CPY*-HA, Cen plasmid, URA3]* | This study |
| MNY3425 | *MATa, leu2-3,112, trp1-1, can1-100, ura3-1, ade2-1, his3-11,15 [UPRE-GFP, TRP1]* | This study |
| MNY3426 | *MATa, trp1-1, can1-100, ura3-1, ade2-1, his3-11,15, leu2-3,112::SFA1-Ole1 [UPRE-GFP, TRP1]* | This study |
| MNY3427 | *MATa, trp1-1, can1-100, ura3-1, ade2-1, his3-11,15, leu2-3,112::SFA2-Ole1 [UPRE-GFP, TRP1]* | This study |
| MNY3428 | *MATa, trp1-1, can1-100, ura3-1, ade2-1, his3-11,15, leu2-3,112::SFA3-Ole1 [UPRE-GFP, TRP1]* | This study |
| MNY3429 | *MATa, trp1-1, can1-100, ura3-1, ade2-1, his3-11,15, leu2-3,112::SFA4-Ole1 [UPRE-GFP, TRP1]* | This study |
| MNY3500 | *MATa his3Δ1 leu2Δ0 met15Δ0 ura3Δ0* | BY4741 |
| MNY3371 | *MATa his3Δ1 leu2Δ0 met15Δ0 ura3Δ0 [pRS313-pADH1-mGFP-mga2(128-1113)]* | This study |
| MNY3372 | *MATa his3Δ1 leu2Δ0 met15Δ0 ura3Δ0 [pRS313-pADH1-mGFP-heh2(93-317)-mga2(128-1113)]* | This study |
