# Supplementary material for: Decoding the impact of lipid saturation on ER signaling networks, ERSU, UPR, and ERAD
Source: PLoS One. 2026 Aug 14;21(8):e0345910. doi: 10.1371/journal.pone.0345910 (PMC13475824; doi:10.1371/journal.pone.0345910)
Supplement: Supplementary file 2 [file pone.0345910.s007.docx]

**Table S2: Plasmids used in this study.**

| ER LipSat sensor | *pRS313-pADH1-mGFP-mga2(128-1113)* | Alwin Kohler lab |
| --- | --- | --- |
| INM LipSat sensor | *pRS313-pADH1-mGFP-heh2(93-317)-mga2(128-1113)* | Alwin Kohler lab |
| pRH1377 | *YCp-CPY*-3xHA, URA3* | Randy Hampton lab |
| pRH2058 | *pPGK-Ste6-166-HA-GFP, 2u plasmid, URA3* | Randy Hampton lab |
| pRH2997 | *UPRE-GFP*, TRP1 | Randy Hampton lab |
| pRH469 | *YIplac211-pTDH3-Hmg2-GFP, URA3* | Randy Hampton lab |
